# Performance of a modular robotic cluster matches skilled human operators for complex cell therapy manufacturing tasks

**DOI:** 10.1016/j.ijpx.2026.100631

**Published:** 2026-08-04

**Authors:** Sudeshna Sadhu, Brigitte Schmittlein, Angela Lares, Christopher Cheng, Xiaojie Chen, Varun Bhatia, Winston Zha, Sebastien Wah, Sunaina Nayak, Marco Uboldi, Yifan Yu, Jiawei Wang, Wei Zhang, Prajakta P. Bhanap, Yongchang Ji, Andrew Scheffler, John Wilson, Dan Welch, Nikolaos Gkitsas-Long, Aidan Jeffrey Retherford, Ramya Tunuguntla, Alice Melocchi, Alexandre Girard, Federico Parietti, Steven A. Feldman, Jonathan H. Esensten

**Affiliations:** a Multiply Labs, San Francisco, CA, USA; b Sezione di Tecnologia e Legislazione Farmaceutiche “M. E. Sangalli”, Dipartimento di Scienze Farmaceutiche, Università degli Studi di Milano, IT; c GenScript Biotech Corporation, Piscataway, NJ, USA; d Thermo Fisher Scientific, Carlsbad, CA, USA; e ScaleReady, New Brighton, MN, USA; f Wilson Wolf Manufacturing Corporation, New Brighton, MN, USA; g Center for Cancer Cell Therapy, Stanford Cancer Institute, Stanford University School of Medicine, Stanford, CA, USA; h Département de génie mécanique, Faculté de génie, Université de Sherbrooke, Sherbrooke, CA; i The Advanced Biotherapy Center (ABC), Sheba Medical Center, Tel Hashomer, IL

**Keywords:** Robotic cluster, T cells, Cell isolation, Harvest, Cell counting, Resuspension, Sampling

## Abstract

Although numerous cell and gene therapies have received regulatory approval, their adoption has been hampered by high cost and challenges in scaling out manufacturing. Many autologous cell therapies are individually manufactured for each patient using traditional manual methods in high-cost environments. Therefore, robotics and automation offer a potential solution to meet the growing demand for such therapies. To ensure identical biological outcomes, automation must replicate validated manual workflows, a requirement that poses significant engineering challenges, especially for aseptic manipulation and compatibility with manual-centric instruments and consumables. Here we describe the design and performance of a modular robotic cluster consisting of specialized modules containing widely adopted equipment. The robotic arm uses custom end-effectors to handle standard consumables and instruments such as syringes, vials, bags, cell counters, bioreactors, incubators, and closed centrifuges. We compared the performance of skilled human operators against the robotic cluster across multiple tasks: transferring cells between sterile bags, cell counting, drawing volume from a vial to a syringe, and resuspension and sampling from both a bag and G-Rex 100 M-CS bioreactor. The robotic system also executed high-complexity operations with industry-standard instruments: cell isolation using a CytoSinct 1000 and wash/buffer exchange with a CTS Rotea Counterflow centrifugation system. Experimental results for each unit operation show that the robotic cluster performance is equivalent to manual operations on the selected key metrics. These data demonstrate that the robotic system can efficiently and robustly perform specific unit operations, which may provide a foundation for future integrated end-to-end cell therapy manufacturing processes.

## Graphical abstract

**Figure:**
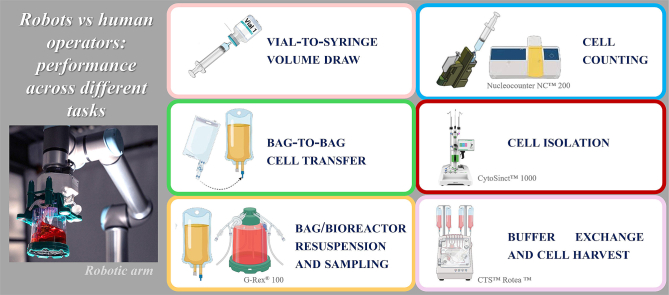

## 1 Introduction

Cell therapy manufacturing is a complex multistep process involving cell isolation, activation, genetic modification, expansion, formulation, and quality control to generate living medicinal products for clinical use (Rieske et al., 2026; June et al., 2014; Costa et al., 2025). Among advanced therapy medicinal products (ATMPs), genetically engineered T-cell therapies have become one of the most successful clinical applications, producing excellent outcomes in hematological malignancies. More recently, these therapies have expanded toward solid tumors, while their application to autoimmune diseases is rapidly emerging (Chung and Kochenderfer, 2024; Schett et al., 2024; Schett et al., 2026; Vukovic et al., 2024). Several autologous T cell products, including chimeric antigen receptor (CAR) T cells, T cell receptor-engineered (TCR) T cells, and more recently tumor-infiltrating lymphocytes (TILs), have recently gained regulatory approval, establishing adoptive T cell therapy as a clinically validated treatment modality (De Marco et al., 2023; Fugger et al., 2020; Greco et al., 2024; Gustafson et al., 2023; Park and Kwok, 2022; Wang et al., 2023; Dias et al., 2024; Song et al., 2022).

These therapies can be manufactured either using autologous or allogeneic cells. While allogeneic approaches hold considerable promise because they offer the potential for off-the-shelf products manufactured in large batches, they still face important biological and clinical challenges, including immune rejection and graft-versus-host disease (Depil et al., 2020; Mason and Dunnill, 2009). As a consequence, the majority of commercially approved T cell therapies rely on autologous manufacturing, in which the same patient serves as both the donor of the starting cells and the recipient of the final product. To date, this patient-specific manufacturing paradigm remains the current clinical standard and is largely responsible for the complexity, cost, and limited scalability of current manufacturing processes, making it the primary target for manufacturing innovation (Song et al., 2022; Abou-El-Enein et al., 2021).

The clinical success of engineered T cell therapies has fueled an extraordinary expansion of the field. As of April 2024, more than 1500 CAR-T cell clinical trials had been registered worldwide, highlighting the remarkable growth of adoptive T cell immunotherapy (Mizuno et al., 2022; Kaiser et al., 2015; Cao et al., 2025). Recent regulatory approvals of TCR-T and TIL therapies further demonstrate that the clinical landscape is rapidly expanding beyond CAR-T products alone. Despite these remarkable advances, manufacturing has emerged as a principal bottleneck limiting broader patient access.

Yet the cost of approved T cell therapies remains a major barrier to broad patient access, with per-infusion prices reaching several hundred thousand US dollars, which are driven in large part by labor and production-related cost of goods (Abou-El-Enein et al., 2021; Fiorenza et al., 2020; Harrison et al., 2018). Manufacturing autologous T cell therapies is inherently challenging because each production batch corresponds to a single patient and requires multiple sequential unit operations, including cell isolation, activation, genetic modification, expansion, washing, formulation and cryopreservation, which need to be performed under Good Manufacturing Practice (GMP) conditions (Ayala et al., 2024; Heathman et al., 2015; Iancu and Kandalaft, 2020; Campbell et al., 2015; Lee and Chang, 2024). Unlike conventional biologics, whose production capacity can be increased through scale-up, autologous T cell therapies require scale-out manufacturing, where increasing patient demand corresponds to an increased number of independent manufacturing batches. Therefore, current approaches remain difficult to scale and are unlikely to satisfy future clinical and market demand (Ayala et al., 2024; Heathman et al., 2015; Iancu and Kandalaft, 2020; Wang and Riviere, 2016). Moreover, aseptic production still relies heavily on manual operations performed by highly trained personnel. This labour-intensive strategy places an unsustainable burden on skilled operators and cleanroom capacity, compounded by the high training requirements and turnover of manufacturing operators (Song et al., 2022; Mizuno et al., 2022; Campbell et al., 2015; Lee and Chang, 2024). This manual production strategy limits throughput and increases risk of errors, contamination, and manufacturing failures (Ayala et al., 2024).

Automation has therefore emerged as one of the most promising strategies to improve robustness, reduce manufacturing costs and increase production capacity. However, most automation efforts have focused on individual unit operations rather than integrated end-to-end manufacturing workflows. For example, dedicated automated devices are available for individual unit operations, such as cell isolation, washing, activation, gene transfer, expansion and formulation, but these instruments are typically operated as isolated automation islands that still require manual transfer of cells and of reagents between processing steps (Kaiser et al., 2015; Melocchi et al., 2025).

Based on the available literature, two major automation strategies have emerged for multi-step automation: the all-in-one and the modular approach (Melocchi et al., 2025; Brindley et al., 2013; Doulgkeroglou et al., 2020; Smith et al., 2019; Moutsatsou et al., 2019; Srai et al., 2024). In the former, multiple manufacturing operations are integrated within a single proprietary platform, such as the Prodigy and the Cocoon systems, which have supported manufacturing of CAR-T, TCR-T and other engineered T cell products in both centralized and decentralized settings (Mock et al., 2016; Lock et al., 2017; Fernández et al., 2019; Castella et al., 2020; Palani et al., 2023; Trainor et al., 2023; Yonezawa Ogusuku et al., 2024; Francis et al., 2023). As a further evolution, microfluidic bioreactors allow autologous T cell production within a reduced footprint (Sin et al., 2024). These platforms simplify workflow implementation and hide complexity from the operators. However, all-in-one systems still require skilled personnel for setup, monitoring and intervention, although the level of operator expertise required is substantially lower than for fully manual processing. On the other hand, all-in-one systems generally offer limited flexibility for process customization, technology upgrades, and integration with third-party equipment because hardware and consumables are tightly coupled within proprietary software. Moreover, all-in-one systems generally lack standardized interfaces for connectivity and data exchange, limiting their use in combination with other equipment. By contrast, the modular approach distributes individual manufacturing operations across dedicated instruments that are integrated into a coordinated workflow. This architecture resembles modern industrial assembly lines, in which the item being processed moves from one dedicated station to the next. This strategy supports equipment interchangeability, incremental technology upgrades as new instruments become available, and process customization, while reducing dependence on a single supplier (Melocchi et al., 2025; Srai et al., 2024; Hort et al., 2022). Most importantly, modular workflows reduce the process constraints from the duration of the full process itself to the longest cycle-time of the isolated unit operations. By decoupling individual unit operations, a modular approach ultimately enables parallel processing, resulting in increased throughput capacity of each individual manufacturing line. Due to these advantages, in recent years the modular approach to cell therapy manufacturing has been developed by many groups (Franscini et al., 2011; Kami et al., 2013; Kikuchi et al., 2018; Mantripragada et al., 2020; Murphy et al., 2017; Rafiq et al., 2016; Thomas et al., 2007). Modular systems have been described for adherent-cell products such as mesenchymal stromal cells and induced pluripotent stem cells, whereas comparable robotic modular workflows specifically designed for suspension T cell manufacturing remain comparatively underexplored (Melocchi et al., 2025; Abraham et al., 2021). Even existing modular approaches have certain limitations: for example, manual transfer of cells and reagents between instruments. These transfers introduce operator-dependent variability, contamination risks due to potential breach of the closed fluid path, and interoperability challenges. At the same time, cumulative time and labor of these transfers may reduce the throughput benefits that modularity is intended to provide, impacting rate and scalability of manufacturing (Mizuno et al., 2022; Melocchi et al., 2025; Ball et al., 2018). In addition, relying on equipment from different vendors can also introduce compatibility and interoperability challenges and requires the need for custom interfaces for data integration and process control. Finally, open manual manipulations are often required in the modular approach for critical tasks such as sampling, media preparation, small volume pipetting, and quality control assays (Franscini et al., 2011; Murphy et al., 2017; Fadeyev et al., 2022; Herbst et al., 2023). In this respect, end-to-end robotic automation has the potential to overcome the above mentioned limitations by combining the flexibility of modular manufacturing with automated material handling and centralized process control. Cost-of-goods analyses further suggest that the greatest economic gains are expected from automating connections and transfers between instruments rather than optimizing individual unit operations alone (Brindley et al., 2013; Doulgkeroglou et al., 2020; Smith et al., 2019; McCoy et al., 2020; Lopes et al., 2020; Tomtishen, 2023). Moreover, robotic automated systems enhance productivity per unit area, potentially decreasing labor costs. Taken together, these advantages of modular manufacturing can unlock the broader clinical and commercial potential of T cell-based therapies.

As a preliminary step toward end-to-end automation, in this study we describe the development and performance of a modular robotic cluster capable of executing common T cell therapy manufacturing unit operations in a flexible sequence. The cluster includes multiple instruments within a single robotically automated platform, maintaining a closed manufacturing workflow with minimized human intervention, thus reducing the risk of contamination. Moreover, the cluster supports integration of hardware and software across multiple Good Manufacturing Practice (GMP)-validated instruments and consumables, all controlled from a single interface. Although the modular architecture of the robotic system may in principle be adaptable to other cell therapy modalities, the present study focuses specifically on autologous T cell manufacturing process, for which scalable and interoperable automation remains a major unmet need.

We have previously assessed the potential of this modular approach, building a proof-of-concept robotic cluster that used industry-standard equipment to successfully culture CD8+ T cells with comparable cell yields, viability, and identity to conventional manual processing (Melocchi et al., 2024). Building upon the above-mentioned work, the present work expands the automated workflow to include bag-to-bag cell transfer, automated cell counting, drawing volume from a vial to a syringe, cell resuspension and sampling from both a bag and G-Rex 100 M-CS bioreactor, cell isolation using CytoSinct 1000 and wash/buffer exchange using CTS Rotea Counterflow centrifugation system. For each operation, robotic performance was systematically compared with manual performance to evaluate accuracy, reproducibility and robustness.

## 2 Materials and Methods

The robotic workflow for each unit operation is shown in the Supplementary material (Video S1). In the current implementation, the single-use consumables used by the robotic cluster are assembled manually by trained personnel prior to loading into the robotic cluster. As a result, the system operates on pre-assembled consumables rather than performing the assembly itself. To minimize misalignment or leakage, assembly occurs following standardized procedures and undergo a quality check before use. Only components that pass this verification are used during automated operation.

### 2.1 Smart-site and Texium connectors

The robotic connectors are based on commercially available Luer connectors, with the addition of external components that increase the robustness of the automated connection/disconnection task (Fig. 2). The standard connectors used during the work are the Beckton-Dickson SmartSite (closed female Luer) and the Beckton-Dickson Texium (closed male Luer). The SmartSite and Texium are closed, needle-free connectors with Luer threads. Both connectors are equipped with a membrane that keeps their default configuration “closed”, preventing any flow through the connector and sealing the internal environment from the outside. The membrane is pushed away into the “open” configuration when a matching Luer lock component is connected and returns to the original “closed” state when the Luer lock component is disconnected. The SmartSite and Texium connectors are classified as Class II, Sterile, Closed System Transfer Devices (CSTD) and approved by the Food and Drug Administration (FDA) for use in clinical/hospital settings (re: K223088 for the SmartSite, and K223076 for the Texium). Needle-free connectors similar to the SmartSite are currently used on several GMP-proven instruments that are employed in the commercial manufacturing of FDA-approved cell therapies, including the G-Rex bioreactor (by Wilson Wolf Manufacturing Corporation). For disinfecting SmartSite and Texium, we utilized the Merit Medical DualCap Disinfection & Protection System. The DualCap Disinfection & Protection System is also FDA approved (re: K142806).

### 2.2 Vial-to-Syringe volume Draw

Each vial for robotic manipulation was attached to a 20 mm vented Vialok (Yukon) and SmartSite (Becton Dickinson) and then assembled to a proprietary vial adapter. Each vial was filled with 5 mL dyed water. For robotic conditions, the two filled vials were placed in the in-house designed vial cartridge. An empty 10 mL syringe with Texium connectors was attached to the coupler and placed in the designed syringe cartridge (Fig. 3a). 4 mL was drawn from vial 1 and 1.8 mL was drawn from vial 2 using the same syringe to reach a target volume of 5.8 mL. Similarly, in the manual condition, 5.8 mL of fluid from two vials was pulled using the 10 mL syringe attached to the connector. The weight of the syringe for both conditions were measured manually and collected pre-draw (tare weight of the empty syringe), post-draw from vial 1, and again post-draw from vial 2 (Analytical Balance, Ohaus).

### 2.3 Bag-to-Bag Cell transfer

Human T cells (StemCell Technologies & AllCells) were seeded into an automation compatible permalife bag (OriGen, PL120-2G) in 100 mL TexMACS media. 24 h post-seeding, cells were activated using TransAct (Miltenyi, 130–111-160). On day 3, cells were transferred from the permalife bag to the transfer pack (Fenwal Inc., 4R2001) robotically and the percentage of cells in each bag was assessed (Fig. 3b).

### 2.4 Bag/Bioreactor Resuspension and Sampling

Robotically enriched and expanded human T cells were harvested on day 8 post-activation from G-Rex 100 M-CS (Wilson Wolf Manufacturing Corporation, J81100-CS) into a 1 L transfer bag (Terumo, BB*T100BB71), resuspended, and sampled for cell counting (Fig. 4a). Post harvest, the cells in the bag were subjected to buffer exchange using the robotic CTS Rotea counterflow centrifugation system. The washed cells were then collected in another 1 L transfer bag, resuspended, and sampled for cell counting. To accomplish robotic bag resuspension and sampling, the robot attached a syringe filled with 10 mL of sterile air to the 1 L transfer bag to flush the tube line. Next, the bag was rocked for 2 min to mix the cells by tilting it up and down at a 30 degree angle from the horizontal position, with the syringe still attached. Following resuspension, the robot mixed the bag line with the syringe five times. A 2 mL sample was then removed from the bag using the same syringe. The sample and bag were then output from the robotic system and cell count was performed. The same 1 L bag was then manually rocked for 2 mins by tilting it up and down at a 30 degree angle from the horizontal position, and a 2 mL sample was manually removed via syringe. All cell counts were performed manually using the NucleoCounter NC-202. This process was repeated on six different 1 L transfer bags, with resuspension and sampling after G-Rex 100 M-CS transfer (*n* = 4) or post-Rotea (*n* = 2).

For the bioreactor resuspension operation, expanded primary human T cells were divided into four experimental conditions in different G-Rex 100 M-CS bioreactors, which included variations in cell number and volume: *i)* low cell number, low volume (5.3 × 10^7^ cells in 100 mL medium), *ii)* high cell number, high volume (1.9 × 10^9^ cells in 600 mL medium), *iii)* low cell number, high volume (4.9 × 10^7^ cells in 600 mL medium), and *iv)* high cell number, low volume (1.3 × 10^9^ cells in 100 mL medium) (Fig. 4b). The robotic cell resuspension protocol was designed to replicate the manual resuspension motion: a scientist from Stanford's Laboratory for Cell and Gene Medicine performed multiple resuspensions with motions recorded and translated to the robotic system using a motion capture system (Opitrak). After each resuspension, all conditions were allowed to rest for 2 h to allow the cells to settle before the next resuspension.

### 2.5 Cell counting

T cells were expanded and transferred into a bag at different concentrations. A 3.5 mL sample was drawn from the bag using a 10 mL syringe (Becton Dickson) by either the robotic arm or a human operator (Fig. 5a). The cells were loaded into a Via1-Cassette (Chemometec, 941–0011). The robotic process used a specialized reservoir to perform the cell transfer from syringe to the counting cassette. Cell counts were performed in triplicate either manually or robotically using NucleoCounter NC-200.

### 2.6 Cell Isolation

Frozen or fresh apheresis was washed with phosphate buffered saline/EDTA (Miltenyi) containing 0.5% human serum albumin and incubated with CD4 and CD8 microbeads (Miltenyi) for 30 min using LOVO Cell Processing System (Fresenius Kabi). The labelled cells were equally divided into manual and robotic conditions for cell enrichment and isolation using the CytoSinct 1000 magnetic cell isolation system. TexMACS media supplemented with 3% serum, IL-7 and IL-15 was used as the buffer for enrichment. For manual conditions, the CytoSinct 1000 TS kit (GenScript Biotech Corporation, D00029) was set up per manufacturer's instructions. For robotic conditions, the kit and bags were assembled onto proprietary cartridges and loaded to the robotic cluster. For both manual and robotic conditions, 2 × 10^9^ target cells were loaded and Enrichment 1.1 protocol was performed. The cell count and viability pre-enrichment were provided by Stanford (Cellometer Auto 2000, Nexcelom Bioscience). Post-enrichment counts were assessed using NucleoCounter NC-202. To quantify T cell purity in the positive fraction, CD3+ T cells were measured by flow cytometry (BD LSR Fortessa, BD Biosciences or CytoFlexLX, Beckman Coulter) performed either at University of California San Francisco (UCSF) flow core or at Stanford University flow core. For each run, separate healthy donor apheresis was used (Fig. 7a).

### 2.7 Buffer Exchange and Cell Harvest

Enriched T cells were expanded and subjected to wash/buffer exchange using the CTS Rotea Counterflow centrifugation system and CTS Rotea Single-Use Kit (Thermo Fisher Scientific, A49585) (Fig. 8a). The published buffer exchange protocol from Thermo Fisher Scientific was performed in both the manual and robotic conditions. However, prime volumes were increased in the robotic condition to account for the additional tubing length. Plasmalyte A containing 4% Human Serum Albumin (HSA) was used as the buffer for both conditions. Cells were harvested in 40 mL of buffer.

### 2.8 Aseptic Process simulation (Media Fill)

Sterile Tryptic Soy Broth media (Corning, MT46060CM) was transferred into pre-sterilized 1 L transfer bags and containers needed for the process simulation in an ISO 5 biosafety cabinet (BSC). The prepared materials were then transferred into the robotic cluster via the input/output module. Here the media was robotically transferred between syringe and G-Rex 100 M-CS bioreactor, from bag to G-Rex bioreactor, and resuspended in the latter. At the end, containers (*n* = 4) were filled with the processed media and checked for sterility in accordance with USP <71>. Post 14-day incubation media growth promotion testing was also performed. To challenge the closed connection capability of the robotic clusters, the above aseptic process simulation was carried out under the worst possible conditions, i.e. having the unit self-contained HEPA/HVAC system purposefully non-operational.

### 2.9 Statistical Analysis

Graphpad Prism 10.4.0. were used to perform statistical analysis. Results are shown as mean ± Standard Deviation (SD) or mean ± Standard Error of Mean (SEM) and statistical differences between manual and robotic conditions were assessed by paired or unpaired Student's *t*-test. A two-tailed *p* value <0.05 indicates a significant difference.

## 3 Results

### 3.1 The modular robotic cluster

**Fig. 1:**
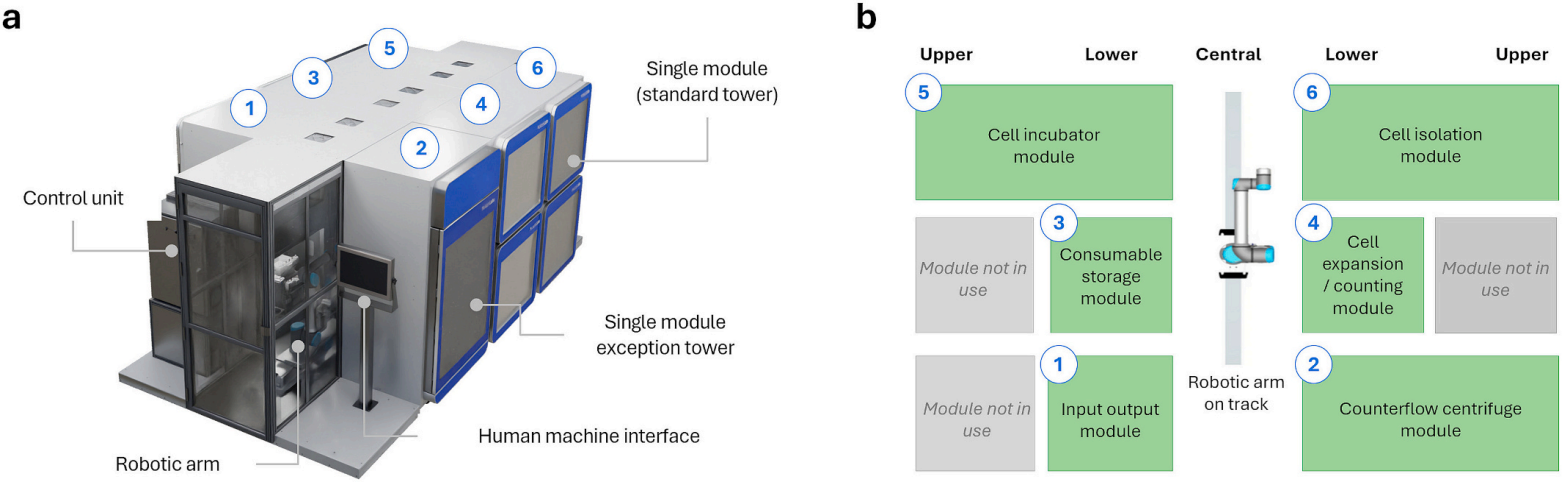
Schematics of the robotic cluster and modules. a) 3D rendered outline of the modular robotic cluster. b) Representative diagram (top view) of the different towers/modules used in this study.

The robotic cluster described in the present work represents an evolution of the system previously described and still represents a research and development prototype (Melocchi et al., 2024). It consists of several modules, which are accessed by a robotic arm mounted on a central rail (Fig. 1a). Each module contains standard equipment or consumables already used in GMP-compliant manufacturing processes. A combination of modules is used to replicate the manual cell therapy manufacturing process. The modules used in the present study are shown in Fig. 1b in green. They have the following functions: the input/output module is for consumable addition and removal from the system. The consumable storage module enables intermediate storage during runs and vial-to-syringe fluid transfer operations. The cell expansion/counting module contains hardware necessary for reagent transfer, cell transfer between bags and bioreactors, and sampling. Cell isolation using the CytoSinct 1000 magnetic cell isolation system (GenScript) and cell harvest using CTS Rotea Counterflow centrifugation system (Thermo Fisher Scientific) were performed in the cell isolation and counterflow centrifuge modules, respectively. The cell incubator module contains a cell culture incubator **(**Thermo Fisher Scientific, HERACELL VIOS 250i**)** capable of storing multiple G-Rex 100 M-CS bioreactors (Cytiva) or cell culture bags.

The development of the robotic system hardware and software was guided by principles including unaltered reproduction of manual bioprocess, easy equipment/consumable validation against manual bioprocess, sterility, modularity, autonomy, traceability, robustness, and usability (Supplementary Material, Table S1). The core principle of our automation strategy is to precisely replicate the validated manual process. By replicating the manual manufacturing process, we can directly transfer a validated manual process to the automated system while minimizing or eliminating major process modifications and extensive comparability studies. The robotic system's design directly implements this principle in two key ways. 1) The system is engineered to use commercially-available consumables (bags, vials, syringes or bioreactors) that are used in manual protocols, ensuring cells only contact materials already validated and the robotic manipulations are designed to emulate their manual counterparts to maintain equivalent process conditions (flow rate, shear stress, temperature, process timing). For some tasks, motions from a human operator were recorded and translated directly to the robot to ensure the system achieves a comparable or equivalent performance to a trained technician. 2) Another design principle is that the entire process is physically isolated from the external environment to minimize the risk of microbial contamination. Manual operations in a cleanroom rely on a variety of aseptic techniques such as working within a biosafety cabinet or using sterile welds for fluid transfer. With these manual techniques, prevention of microbial contamination is dependent on the skill of the operator. By contrast, the robotic system uses sanitized needle-free connectors for all fluidic transfers. The interior of the robotic cluster has an air filtering and monitoring system to further minimize contamination risk.

A comprehensive summary of the key metrics assessed for each unit operation under manual and robotic conditions is provided in the Supplementary Material (Table S2).

The robotic cluster hardware is designed with modular architecture to accommodate any combination of instruments based on the unique needs of the cell manufacturing process. Modularity is achieved through swapping modules with different instruments or functions, enabled by customizable module deckplates. There are minimum modifications performed to the off-the-shelf instruments and the associated consumable kits for easier and faster technology transfer process from manual to automated workflow. The robotic system software operates autonomously. The only human interaction with the robotic cluster involves loading and unloading consumables via the input/output module. To make manual interventions user-friendly, the cluster process planning and execution software (the “orchestrator”) allocates load and unload waves and displays detailed loading instructions in the user interface. For traceability, every operation performed in the robotic system, including reagent transfer volumes and instrument parameters, is tracked in the digital batch record. The orchestrator's parallel planning algorithm improves manufacturing throughput by enabling a single robotic arm to switch between tasks for different batches, taking into account pre-defined biological time constraints.

### 3.2 Automated liquid handling approach

Closed connections between different vessels in traditional manual processes are typically done via tube welding, which is difficult to automate. Welding entails fitting flexible tubes into grooves on a metal plate, locking the tubes into place, pressing a button on the welding instrument, removing the tubes, and squeezing the weld site to open the interior fluid path. As an alternative for the robotic cluster, we developed rigid protected connectors (Melocchi et al., 2024).

Rigid connectors enable reliable connections and address all the disadvantages of tube welding. Operating rigid connectors based on a standardized thread (e.g. Luer lock) is straightforward for both human operators and robotic systems. A successful connection can be confirmed by measuring simple process parameters such as locking torque [Nm] or linear advancement along the thread [mm]. The connectors are designed to work together as a system. The sterile fluid path is closed unless connectors are mated to each other. Rigid connectors can be attached to known lengths of tubing and fixed to specified locations within the robotic cluster. This approach allows the robotic arm to find and utilize the connector at its fixed location.

**Fig. 2:**
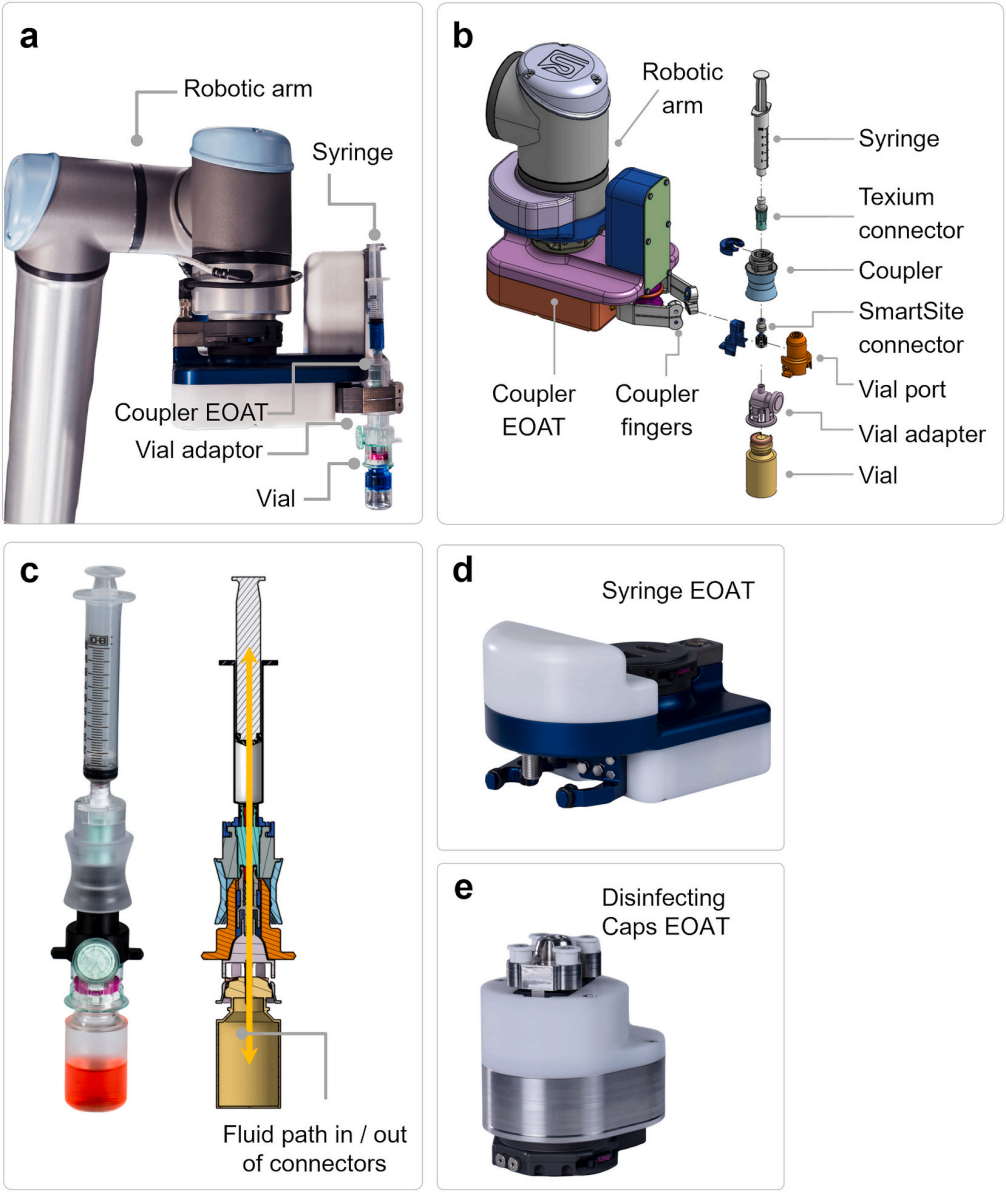
Liquid handling automation tools. (a) Image of the 6 axis robotic arm with a coupler EOAT grasping a vial and syringe assembly. (b) Exploded view diagram of the robotic vial and syringe assembly. (c) Section view for the connector assembly showing liquid flow path. (d) Image of the syringe EOAT. (e) Image of the disinfecting cap EOAT.

One example of this approach is fluid transfer between a syringe and a vial (Fig. 2). A customized port and coupler enable the robot to perform this process (Fig. 2a,b). The port is a snap-on plastic adapter that surrounds a SmartSite needle-free female luer connector. The coupler is a snap-on plastic adapter that surrounds a Texium needle-free male luer connector. These proprietary adapters do not modify the connector and do not come in contact with the sterile fluid path. The port contains external features that allow the vial adapter to be docked in a fixed location. The port has conical lead-in features that enable the mating coupler to be reliably connected. The coupler has complementary internal lead-in features that guide in onto a Port and external conical features that allow the coupler end of arm tooling (EOAT) (Fig. 2a,b) to grasp the coupler, self-center it within the gripper, and rotate it to engage the luer lock threads.

The robotic arm is equipped with a 6-axis force-torque sensor that enables it to estimate interaction forces and adjust positioning during the coupler-port mating interaction and ensure that the connection is centered. Monitoring for a proper connection is done via several sensors in the system. For example, the force-torque sensor in the robot arm checks that the expected force thresholds are met during the connection process. When the connector is fully engaged and bottoms out, force will spike in the vertical direction past a threshold.

For fluid transfer between a vial and a syringe, the port is attached to the vial adapter, and the coupler is attached to the syringe. When the syringe and vial are mated together, a pathway is opened for fluid to be transferred from the vial to the syringe (Fig. 2c). The coupler EOAT grasps the vial and syringe assembly and allows the robot arm to invert the vial and slot its plunger into a syringe pulling fixture. This inversion allows fluid to be drawn from the vial into the syringe. After this step is completed, the vial is docked back into its cartridge, the syringe is decoupled, and then connected to the target vessel for dispensing. A syringe EOAT (Fig. 2d) is connected to the robot arm and enables the arm to sense where the plunger of a syringe is, grasp it, and push or pull it to dispense or withdraw fluid (Supplementary Material, Video S1). We designed robotic grippers to automatically operate disinfecting caps (Fig. 2e). Disinfecting caps are commercially available, one-time-use components that contain a disinfecting solution and a thread that matches the shape of the SmartSite and Texium connectors. The use of disinfecting caps on the connectors before coupling further decreases the risk of microbial contamination.

### 3.3 Bag-to-bag cell transfer and vial-to-syringe volume draw

**Fig. 3:**
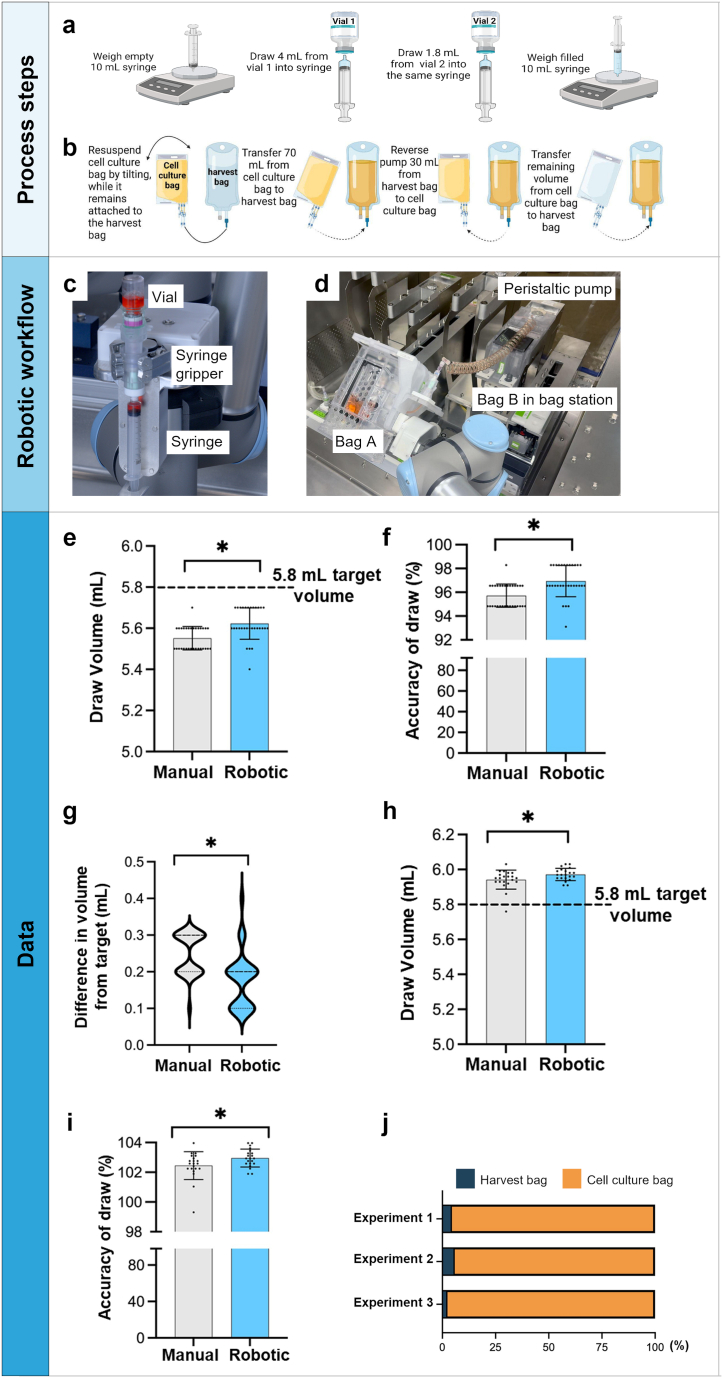
Accuracy of vial-to-syringe draw in manual and robotic conditions and automated bag-to-bag transfer of human T cells. (a-b) Schematics showing the (a) robotic vial-to-syringe draw and (b) robotic bag-to-bag transfer workflow. (c) Image showing robotic arm holding the vial to the syringe assembly in an inverted manner to draw fluid. (d) Transfer of cells from bag A to bag B using a peristaltic pump. (e-g) Data showing initial calibration conditions (mean ± SD, unpaired *t*-test. *n* = 31). (h-i) Data showing modified calibration conditions (mean ± SD, unpaired t-test, *n* = 20). (j) Data is represented as the percentage of cells in each bag after the transfer was complete. n = 3 independent experiments.* indicates statistically significant, *p* < 0.05.

Withdrawal of liquid from a reagent vial with a syringe (Fig. 3a) and bag-to-bag transfer of liquid (Fig. 3b) are routine tasks in cell therapy manufacturing. The underlying design principles of the automated vial-to-syringe interaction were described above. The accuracy of a vial-to-syringe fluid draw was assessed using standard glass vials and 10 mL syringes. The consumable module in the robotic cluster includes a vial and syringe cartridge dock used for storage and manipulation of vials and syringes as well as a syringe puller. The robotic gripper picks the syringe coupler, places it on top of the vial and locks the vial and syringe port together by engaging the Texium connector onto the vial (Fig. 3c). The vial-syringe assembly is then inverted and the barrel flange of the syringe is constrained to a fixed location on the syringe puller. The robotic gripper then pulls the syringe plunger to a specific distance for drawing the fluid.

For transferring liquid between two bags, we designed an in-house bag station consisting of load cells, peristaltic pumps and a dock for bag cartridges (Fig. 3d). Off-the-shelf bags were custom-modified by attaching a coiled pump tube, ending in a Texium connector at one end and a SmartSite needle-free connector at the other. The modified bags were assembled on a bag cartridge compatible with robotic handling. The bag cartridge is aligned automatically with a peristaltic pump which allows fluid to be pumped to and from a bag in either direction. To achieve precise bag-to-bag liquid transfer, the software of the robotic cluster monitors the change in bag weight with load cells that were integrated within the bag station. Based on the change in bag weight, we computed the real time flow rate (change in bag weight over time) and dynamically adjusted the pump motor speed to accurately pump to the set target volume.

To compare side-by-side manual vs robotic vial-to-syringe draw accuracy, an arbitrary target volume of 5.8 mL was chosen as the total draw volume from two vials. The average draw volume was 5.55 mL for the manual condition and 5.62 mL for the robotic condition (Fig. 3e). The accuracy was slightly higher in robotic draws compared to manual (Fig. 3f). There was considerable overlap in the volume difference from the target volume between the manual and robotic conditions (Fig. 3g). We calibrated the robotic system to account for dead volume in the syringe hub and performed an additional experiment. The target volume remained 5.8 mL. The average volume for the manual conditions was 5.94 mL and for the robotic condition 5.97 mL (Fig. 3h). The CV was 0.91% for the manual condition (*n* = 21) and 0.58% for the robotic condition (n = 21). Both the manual and robotic conditions were able to achieve a draw volume slightly above the target volume (Fig. 3i). To validate the robotic bag-to-bag transfer unit operation, we assessed the efficiency of cell transfer between bags. We cultured human T cells in 100 mL growth media in a source bag (bag A) and transferred the entire cell suspension to the final transfer bag (bag B) using a closed-loop pumping system with intermittent robotic resuspension of the source bag. Post robotic bag-to-bag transfer, we assessed the distribution of cells in both the source and the final bag. Across three experiments, an average of 96% of viable cells were successfully transferred, with 4% viable cells retained in the initial bag (Fig. 3j).

### 3.4 Bioreactor or Bag Resuspension and Sampling

**Fig. 4:**
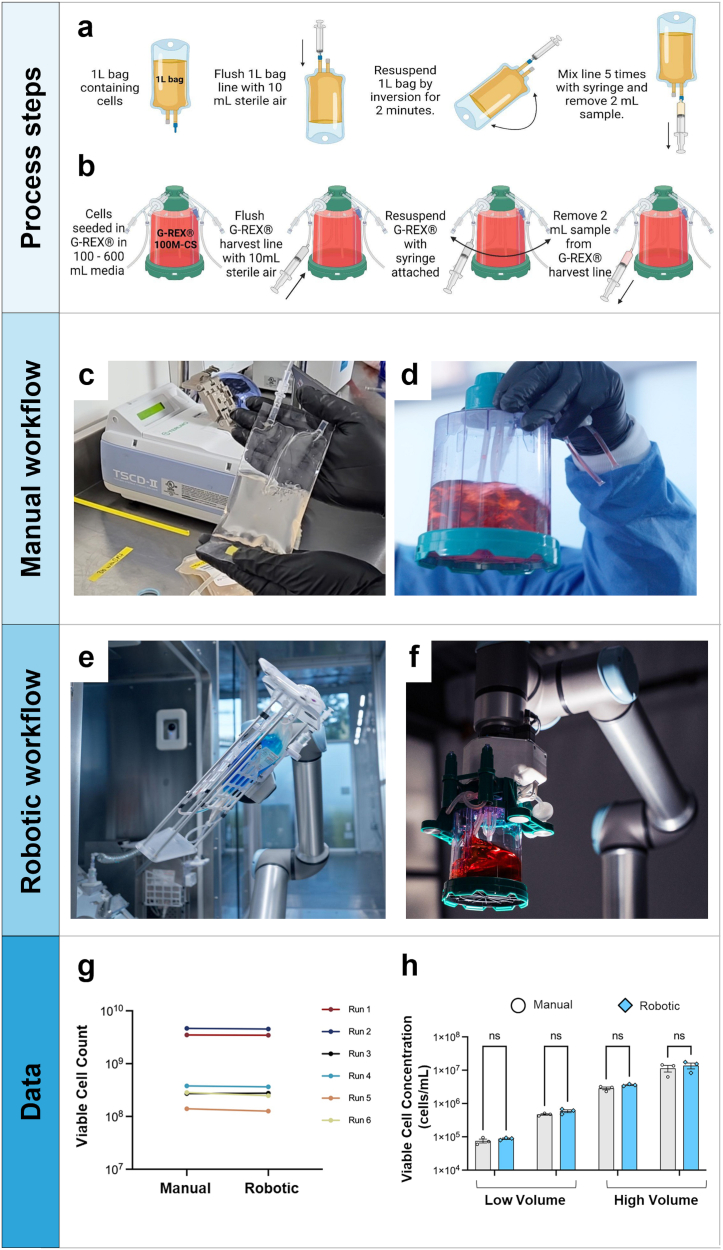
Manual versus robotic resuspension of cells in a bag and bioreactor. (a,b) Schematics showing robotic process steps for 1 L transfer bag and G-Rex 100 M-CS bioreactor resuspension and sampling respectively. (c,f) Image of bag and bioreactor resuspension performed manually (c,d) and robotically (e-f). (g,h) Manual versus robotic resuspension of human T cells in a 1 L transfer bag and G-Rex 100 M-CS bioreactor. Viable cell counts post bag resuspension were measured (*n* = 6). (g) Cells at different concentrations and volumes in a G-Rex 100 M-CS bioreactor were resuspended with manual or robotic swirling motion. (h) Cell concentration was then measured (mean ± SEM, multiple unpaired *t*-test, *n* = 3 per condition).

Cells tend to settle or clump together in the bags or bioreactors, leading to uneven distribution in the vessel. Mixing of the vessel uniformly distributes the cells, ensuring that subsequent samples are representative of the culture contents. In this unit operation, we manually or robotically mixed a bag or a bioreactor, sampled using a syringe, and measured the resulting cell concentration (Fig. 4a-d). Bag mixing is automated through a simple robotic trajectory involving the 6th joint on the robot (Fig. 4e). The trajectory is defined such that the robot's tool center point rotates 180 degrees about the tool center point axis. In contrast, mixing of cells in a G-Rex bioreactor was automated by capturing an experienced scientist's hand motions using the motion capture system OpiTrak. This motion was then extracted and converted into the base frame of the robot, enabling the robot to precisely mimic these movements when holding a G-Rex (Fig. 4f). This approach can be extended to automate any custom mixing motions. To automate syringe sampling from a bag or bioreactor, the software communicates with a proximity sensor that is built into a custom end-effector of the robot. The software takes into account the conversion between robot tool center point movement in mm and syringe plunger displacement in mL to a precision of 0.1 mL. This approach allows the robot to accurately detect where the syringe plunger is and how much to pull to draw a particular volume of sample provided by the operator.

We experimentally compared the performance of manual and robotic mixing processes. One liter transfer bags or a G-Rex 100 M-CS bioreactor were assembled into proprietary robotic cartridges. Sampling was performed using either a 5 mL or 10 mL syringe assembled on a robotic coupler (Fig. 4c,d). For comparing manual versus automated bag mixing, primary human T cells at low (1 × 10^6^ cells/mL) and high (20 × 10^6^ cells/mL) concentrations were placed in 50–300 mL volumes in 1 L bags. The bags were mixed by manual or robotic mixing for 2 mins. The bags were then sampled using a syringe and counted (Fig. 4a). On average, across six runs, the cell counts after robotic mixing were within 5.4% of the manual process average counts (Fig. 4g). To evaluate manual and robotic G-Rex mixing, primary human T cells in two volumes (100 versus 600 mL) and a range of cell concentrations (0.08 × 10^6^ - 13.7 × 10^6^ cells/mL) were placed into a G-Rex 100 M-CS bioreactor (Fig. 4h). The cells were allowed to settle for 2 h and then resuspended manually or robotically with a swirling motion. Cells were then sampled and counted. Viable cell concentrations obtained by manual and robotic resuspension were not significantly different across all four conditions tested (Fig. 4h), demonstrating similar cell counts between the two mixing methods across the full range of volumes and cell concentrations evaluated.

### 3.5 Cell counting

**Fig. 5:**
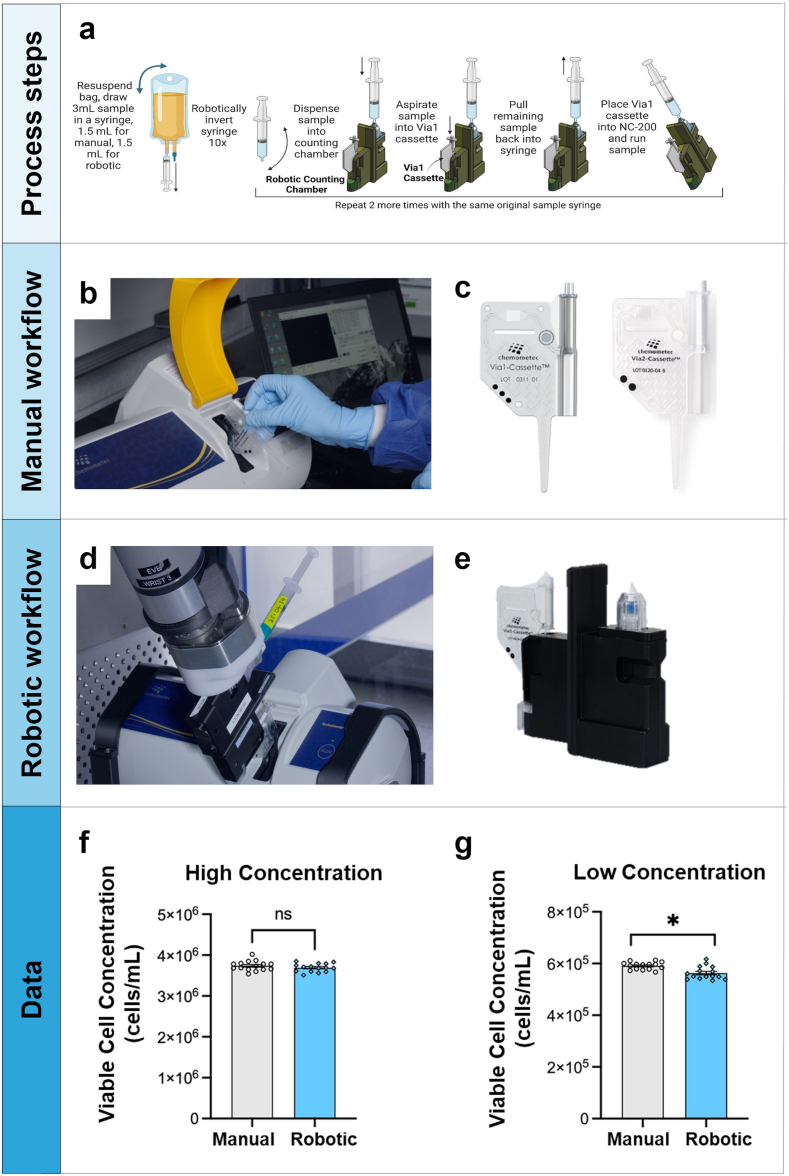
Comparison of manual versus robotic cell counts at high and low concentrations using a NucleoCounter NC-200 cell counter. (a) Schematics showing robotic cell counting workflow. (b-c) Image of (b) operator performing cell count and (c) off-the-shelf Via-1 cassette used in manual workflow. (d-e) Image showing robotic arm inserting the robotic cassette in NucleoCounter NC-200 for cell count. (f-g) Data showing manual and robotic comparison of viable cell concentration at high concentration (left) and low concentration (right) (mean ± SEM, unpaired *t*-test, for each concentration, n = 15 cell counts for manual and n = 15 cell counts for robotic). * indicates statistically significant results, *p* < 0.05.

Cell counting and viability assessment is performed at many points of the cell therapy manufacturing process. Cell counting is manually performed by drawing a small volume of liquid (cell suspension) from a bioreactor or cell culture bag. The liquid is loaded into a cassette which is inserted into a cell counting instrument. We mimicked these manual process steps robotically (Fig. 5a-c). The cell counting module uses an automated NucleoCounter NC-200 instrument with a custom designed cartridge to house three robotic cassettes (Fig. 5d,e). Each robotic cassette has a fluidic chamber where the cell suspension is dispensed and temporarily stored. Then a cell counting cassette containing immobilized staining solution is dipped in the fluidic chamber and cell suspension is drawn in the cassette. To start a cell counting protocol on the NC-200, the robot uses a custom end-effector which allows it to press the “RUN” button on the instrument. Once the cell count is complete, the software acquires the cell count files from the counter using the SMB file sharing communication protocol. These results are then stored and shared with the operators within the digital record as soon as they are retrieved.

To compare side-by-side the manual and robotic cell counting, human T cells were placed in bags at two concentrations: 3.5–4.0 × 10^6^ cells/mL (“high”) and l5.3–6.1 × 10^5^ cells/mL (“low”). Using 10 mL syringes, a small volume of cell suspension (∼3 mL) was drawn from the bag and cells were counted on a NucleoCounter NC-200 by either a manual operator or a robotic process. The cell concentrations measured were comparable between manual and robotic approaches. For the high concentration, the average result was 3.74 × 10^6^ cells/mL (manual, *n* = 15) and 3.69 × 10^6^ cells/mL (robotic, n = 15). The coefficients of variation (CV) were also similar: 3.23% (manual) and 2.77% (robotic). Results were also comparable at the low concentration although a statistically significant lower count for the robotic condition was observed: 5.91 × 10^5^ cells/mL (manual, n = 15) and 5.63 × 10^5^ cells/mL (robotic, *n* = 15). The CV was also comparable: 2.41% (manual) and 4.47% (robotic) (Fig. 5f). The slightly higher variability observed for the robotic condition at low cell concentration could be attributed to several factors: longer interval between robotic sampling and counting, small sample volume, low number of replicate counts, and lack of mixing immediately prior to sampling. We plan further optimization of the sampling and counting procedure at low cell concentrations.

### 3.6 Cell Isolation

**Fig. 6:**
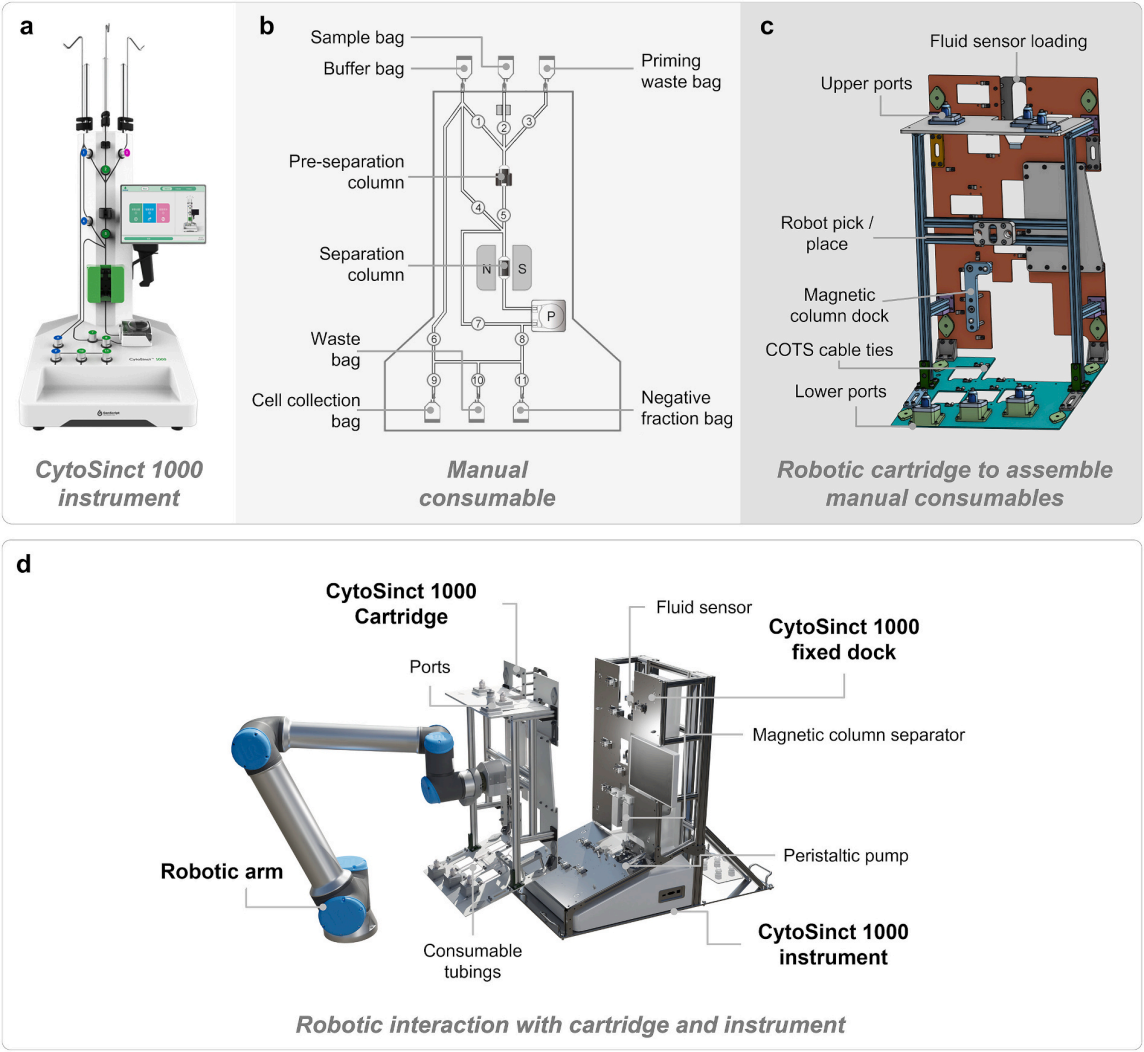
Automation strategy for CytoSinct 1000 magnetic cell isolation instrument. (a) Image of off-the-shelf instrument used in manual workflow. (b) off-the-shelf standard consumable used in manual cell isolation workflow. (c) Cartridge to assemble standard consumable for automated process. (d) 3D render showing different components for instrument automation: robotic arm interacting with robotic cartridge and off-the-shelf instrument with dock plate to house robotic cartridge.

Cell isolation is performed to obtain a purified population of target cells, such as T cells, from the heterogeneous cell population present in blood. In the robotic system, we incorporated a CytoSinct 1000 automated cell isolation platform. The instrument is designed to isolate specific cell types from a heterogeneous cell population using an immuno-magnetic cell isolation process (Fig. 6a). The key components of the instrument include a liquid sensor to monitor the entry of sample into the consumable kit, a magnetic separation unit, a peristaltic pump to control the fluid flow rate, and pinch valve to control flow direction within the kit (Fig. 6b). Proper integration and positioning of the components present on the consumable kit onto the instrument are essential for successful cell isolation. The cell isolation module was automated to meet the following requirements: the instrument uses a standard consumable, the module autonomously executes protocols, and the performance is equivalent to a system run manually.

The robotic CytoSinct consumable kit was added onto the instrument utilizing the CytoSinct 1000 cartridge (Fig. 6c). There were two major challenges associated with the consumable loading task. First, the cartridge has a tall profile, rendering accurate pick/place difficult due to risk of cantilevering and structural deflection. To address this challenge, the robotic pick/place interface is located at the cartridge's center of mass (Fig. 6c). Both planes of the cartridge were roughly aligned with the CytoSinct 1000 fixed dock (Fig. 6d) using slots that mate with alignment pins on the dock. After loading, electromagnets are engaged to hold the cartridge in place. The second load challenge was that the CytoSinct 1000 instrument has eleven different pinch valves requiring lateral tube insertion. An augmented valve approach was thus developed to support front-loading of the tubes. A break-beam sensor detected whether the instrument's valve was closing, and the augmented valve closure followed within milliseconds. To communicate with the instrument without requiring any usage of the touchscreen by the operator, a custom Application Programming Interface (API) was developed in collaboration with the manufacturer, GenScript. Specifically, the software can communicate with this API using the Transmission Control Protocol/Internet Protocol (TCP/IP) communication protocol, allowing the operators to bypass any steps requiring the touchscreen on the instrument and run any custom protocol.

**Fig. 7:**
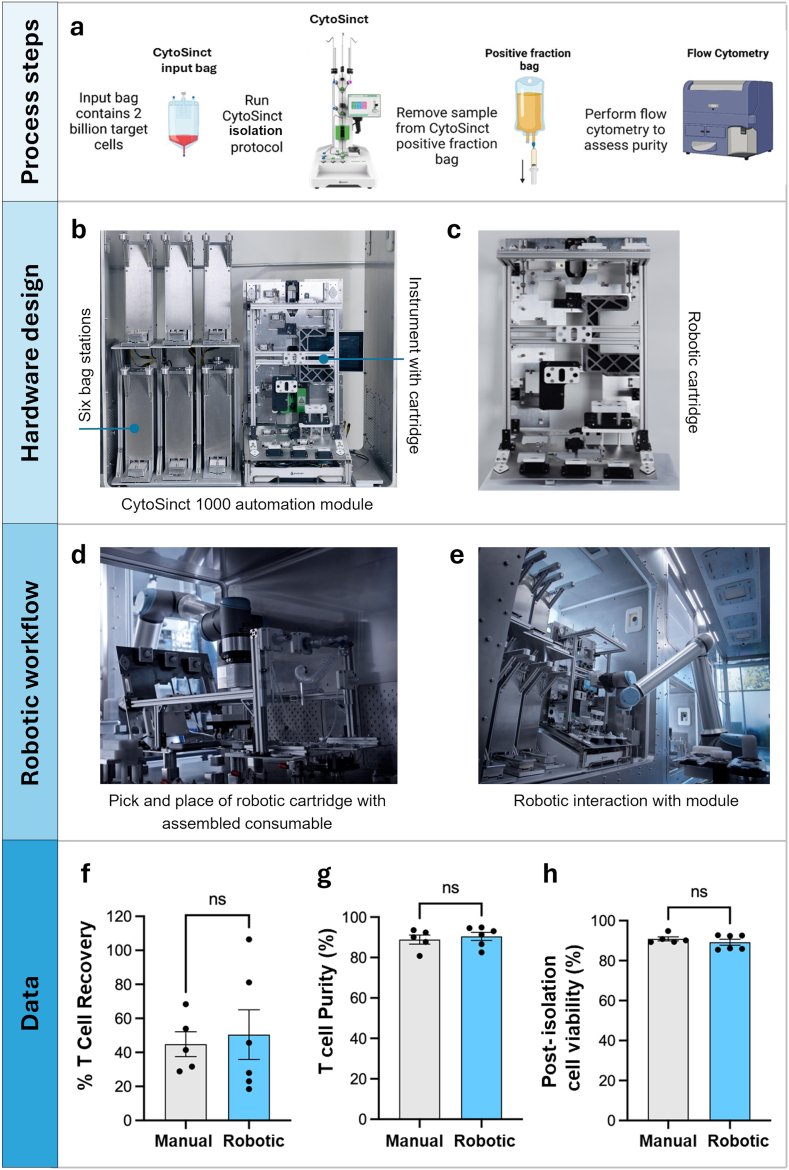
T cell recovery and purity comparison between manual and robotic cell isolation using a CytoSinct 1000 magnetic cell isolation system. (a) Schematics of the robotic and manual T cell isolation workflow. (b) Image showing the CytoSinct module housing the automated instrument with cartridge assembled and six bag stations. (c) Image of the robotic cartridge on which off-the-shelf consumable is assembled. (d) Image showing robotic arm picking up robotic cartridge with consumable assembled from input/output module. (e) Robotic arm interacting with the CytoSinct module. (f) Percent recovery of T cells in the positive fraction compared to the starting material. (g) post-isolation T cell purity in the positive fraction, and (h) post-isolation cell viability. Data shown as mean ± SEM. Statistical comparisons are an unpaired *t*-test, ns = non-significant. n = 5 for manual condition and n = 6 for robotic condition.

Cell isolation on the CytoSinct is performed by labelling a heterogeneous cell suspension with magnetic beads that bind specifically to T cells. The labelled cells are then processed on the CytoSinct 1000 instruments using the TS consumable kit (Fig. 7a). The cell isolation module (Fig. 1b) contains a CytoSinct 1000 magnetic cell isolation system and bag stations (Fig. 7b). An off-the-shelf CytoSinct 1000 Tubing Set was modified by adding tubing with a SmartSite port to enable closed system connection with the bags and assembled on the robotic cartridge (Fig. 7c). The relevant bags were custom designed to be compatible with the robotic loading or unloading onto the enrichment/isolation module bag stations. The robotic cartridge and bags were introduced into the robotic system via the input/output module and the robotic arm shuttled it to the enrichment/isolation module (Fig. 7d). Once the robotic cartridge with tubing kit is docked and all the key components are robotically placed on the instrument, the relevant bags are connected to the tubing kit and cell isolation protocol is initiated (Fig. 7e).

We performed a concurrent manual (*n* = 5) and robotic (*n* = 6) positive isolation of T cells using a CytoSinct instrument and the CytoSinct 1000 TS tubing kit. Frozen leukapheresis containing heterogeneous cells was thawed, washed, and incubated with CD4 and CD8 magnetic microbeads. The target cell input range was 0.5 × 10^9^–2.0 × 10^9^ cells. Post-isolation, the total T cell yield recovery was comparable between manual and robotic conditions, although greater variability was observed in the robotic condition compared to manual (Fig. 7f). Post-isolation T cell purity was assessed by flow cytometry (Fig. 7 g). The percentage of CD3+ T cells in the positive fraction was comparable between conditions, and above 80% in all runs. Post-isolation cell viability was quantified in the positive fraction for both the manual and robotic processes (Fig. 7 h). Comparable results for cell viability were observed in both processes. Other cell subsets were also quantified (Supplementary Material, Fig. S1).

### 3.7 Buffer Exchange and Cell Harvest

The CTS Rotea is a closed automated counterflow centrifugation system that has several functions, including cell separation based on size and density, volume reduction, cell wash, and buffer/media exchange (Supplementary Material, Fig. S2a). To perform these functions, a closed single-use sterile consumable kit (Supplementary Material, Fig. S2b) is used. The instrument is composed of several key components: a peristaltic pump for fluid movement, valves to direct fluid flow, a centrifuge cone for density-based separation of fluid contents, and various pressure and bubble sensors that act as step triggers and aid in troubleshooting. Before operating the instrument, various bags are welded to the consumable kit's tubing. Different protocols are then executed on the instrument, depending on the specific application.

The CTS Rotea consumable kit was mounted onto the instrument using a custom aluminum cartridge, designed to enable robust, repeatable handling by the robotic arm. The cartridge was seated onto the fixed dock on the instrument via locating pins, then secured in place using two cams, one on each side of the instrument (Supplementary Material, Fig. S2c,d). Following the large-scale docking of the full consumable cartridge onto the Rotea platform, several smaller, precise loading steps were required to complete setup. The pump load mechanism (Supplementary Material, Fig. S2d) uses a specific pump motor to transcribe a circular arc to fully seat the tubing in the peristaltic pump. The centrifuge cone was placed into its chamber using an integrated alignment feature on the CTS Rotea cartridge itself, ensuring precise positioning. For centrifuge cone removal, a photoelectric sensor on the instrument's centrifuge chamber clip detected the cone's orientation, and the cone included a dedicated robotic gripper interface for independent extraction based on orientation. To ease insertion of each tube into its respective bubble sensor, a pre-load compression jig was used to stretch the tubing, reducing its diameter for smoother engagement with each bubble sensor's clamshell-shaped tube housing. Foam inserts were added between the centrifuge cone and the robotic cartridge to eliminate rattling during high-speed centrifugation steps.

**Fig. 8:**
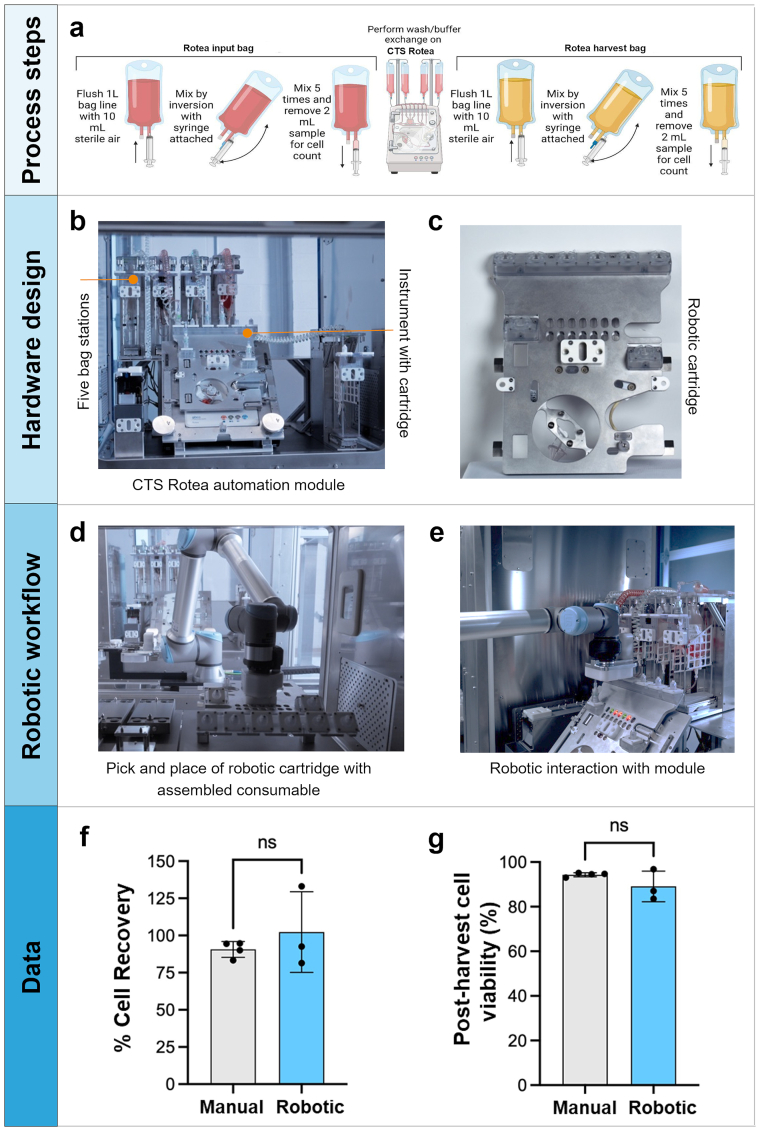
Comparison of Manual and Robotic Harvest Processes Using the CTS Rotea Counterflow Centrifugation System. (a) Schematics of the robotic and manual harvest workflow. (b) Image showing the CTS Rotea module housing the automated instrument with cartridge assembled and five bag stations. (c) Image of the robotic cartridge on which off-the-shelf consumable is assembled. (d) Image showing robotic arm picking up robotic cartridge with consumable assembled from input/output module. (e) Robotic arm interacting with the CTS Rotea module. (f) Data showing percentage cell recovery, and (g) post-harvest total cell viability between manual and robotic T cell harvest (mean ± SEM, unpaired student's *t*-test, non-significant difference between manual and robotic condition. *n* = 4 for manual and *n* = 3 for robotic).

To confirm the performance of the automated Rotea, we performed cell washing and buffer exchange both manually and using an automated (robotic) process. Cells were harvested into a 1 L bag, and both the input cell bag and reagent bags were loaded into either the manual or robotic Rotea system. Cell recovery and viability were assessed by performing cell counts before and after the Rotea process (Fig. 8a). For the robotic process, the counterflow centrifugation module (Fig. 1b) consists of bag stations, a robotic cartridge for holding the modified consumable kit and a dock for holding the Rotea instrument (Fig. 8b,c). The consumable kit is first loaded into the input/output module, then transferred to the centrifugation module using a robotic cartridge gripper (Figs. 8d, e). Once correctly installed, the fluid bags are automatically connected to the consumable kit positioned on the instrument. We compared the cell recovery and manual versus robotic buffer exchange using the Rotea and primary human T cells (ranging from 0.2 × 10^9^ to 4.0 × 10^9^) in independent runs. The cell recovery for the manual process ranged from 83% to 95%, while the cell recovery for the robotic process ranged from 81% to 133% (Fig. 8f). The cell viability post-harvest was above 93% in the manual condition, whereas in the robotic condition, viability ranged from 83% to 97% (Fig. 8g). There was no significant difference in percent cell recovery or post-harvest cell viability between manual and robotic conditions.

### 3.8 Aseptic Process simulation (Media Fill)

To confirm the ability of the robotic system to maintain sterility during cell manipulations, aseptic process simulations were carried out for a model CAR-T cell manufacturing process. In the media fill experiment, the unit operations were performed sequentially and included transfer of volume between syringe and G-Rex bioreactor, transfer of volume from bag to G-Rex bioreactor, and resuspension of G-Rex bioreactor, overall representing over 350 connections. No bacterial growth was observed in the media after 14 days of incubation at 37 °C or longer (Supplementary Material, Table S3). Notably, the robotic system was found to maintain sterility of the process when placed in a standard research laboratory space (non-classified environment).

## 4 Discussion

In this work we described a modular, research-and-development prototype robotic cluster able to perform several common cell-manufacturing unit operations. Results were similar to those of a trained human operator, using non-adherent primary human T cells as a representative and industrially relevant cell type. The system reproduced complex manual processes with the same standard equipment and consumables and, where required, could mimic human motion. For example, resuspension in a G-Rex 100 M-CS bioreactor was enabled by motion capture of a human operator. The aim was not to identify or validate an optimal motion pattern but to establish a well-defined, reproducible reference. The movements of a single experienced operator were recorded so that the robot could reproduce them. No metric was defined to score the quality of those movements. The manual protocol therefore served as a practical, established benchmark for validation rather than as an optimal strategy. Because modelling a single operator neither guarantees optimality nor captures inter- and intra-operator variability, a systematic assessment of operator-to-operator variability, together with quantitative optimization of mixing and resuspension strategies, potentially cell-type-specific and informed by imitation- and reinforcement-learning approaches, will be an important focus of future work.

Automating individual instruments occasionally required software adaptation. To improve cell recovery in the robotic CytoSinct runs, for example, we modified the Enrichment 1.1 protocol by lengthening the washing and elution steps. Comparable adjustments are likely to be needed for instruments that use large, dedicated tubing sets. The versatility of the instruments themselves also broadens the applicability of the approach: the CTS Rotea Counterflow Centrifugation System supports washing, dilution, volume reduction, aliquoting and media exchange, so the automation developed here can be extended to other unit operations performed on the same device. In one Rotea run, cell recovery exceeded 100%, possibly because cell clumps formed during culture were dissociated into single cells during processing.

Several operations are not yet automated. For example, we did not characterize the precision and accuracy of partial large-volume transfers (>100 mL) or cell-transfer efficiency between vessels. Small volumes (<1 mL) remain challenging, requiring careful calibration for viscosity, surface tension, dead volumes, and consumable geometry. All of the above are targets for further development. The current cluster also lacks dedicated real-time detection of syringe or bag clogging and dedicated leak sensors, although no clogging or leakage occurred during the experiments reported here. Future iterations may add monitoring strategies, such as pressure sensing and flow- or camera-based inspection of critical areas, to flag abnormal conditions and trigger corrective actions. On the software side, the orchestrator software provides the operator interface and records process data in the digital batch record. The software also includes decision-support features that convert in-process measurements such as cell number into actionable triggers such as harvest timing, together with the associated alerting, are a planned extension.

Because every unit operation must preserve product sterility, the cluster relies on multiple-use, needle-free connectors sanitized with alcohol-containing disinfecting caps before each use. All activities take place inside the cluster enclosure without human intervention. Since operators are the principal source of microbial contamination in cleanrooms, the risk of culture contamination is expected to be lower than in manual processing, potentially allowing a less stringent cleanroom classification (Grade D or C rather than Grade B) for the cluster interior. For GMP manufacturing, the interior of the cluster will require defined cleaning and environmental-monitoring procedures; cleaning-in-place and sterilization-in-place were outside the scope of this study, and their extent will be established empirically in future work.

A key advantage of the proposed architecture is its scalability. Multi-arm parallelism and the concurrent processing of multiple patient batches are expected to raise throughput and operational flexibility well beyond that of a single operator. As robotic actions become faster and more reliable, the overall process time will fall further. Because consumable use per run is comparable to the manual workflow, total consumable consumption scales linearly with throughput when the system operates at full capacity. A systematic comparison of the consumables required for each unit operation in the manual and robotic workflows is provided in the Supplementary Material (Table S4). Reaching commercially relevant scale-out will require standardization and cost-efficient mass production of the robotic adapters for each consumable and instrument. Modules for the automated handling of a wider range of consumables are already under development.

Moving toward a fully integrated end-to-end platform will require automating the operations still performed off-line, including immunolabelling, small-volume harvest, formulation, fill-finish and cryopreservation. Future software versions will allow users to customize process parameters, adjust workflows mid-run and integrate different vessels and consumables. Importantly, the automation strategy is deliberately vendor-agnostic: it is built on standard, commercially available consumables and on reconfigurable modules, custom end-effectors and instrument-agnostic software interfaces, including APIs co-developed with instrument manufacturers. The same approach can in principle be transferred to instruments from other vendors with the extent of (re)development depending on each instrument's consumable format and control interface.We are actively expanding the range of supported equipment, prioritizing widely-used and GMP-qualified instruments to avoid vendor lock-in.

Robustness will be reinforced through fault-tolerant control, automated error detection and recovery, and real-time barcode-based traceability of consumables, reagents and samples. Taken together, these measures enable full starting-material traceability and patient chain-of-custody management, a prerequisite for GMP-compliant, clinical-scale deployment that is currently under active development. In parallel, imitation- and reinforcement-learning frameworks, together with digital-twin modelling, are expected to support anthropomorphic motion planning and predictive validation of workflows before live execution.

Beyond lowering the cost of goods, the reduced cleanroom requirement afforded by the modular robotic approach could, in principle, facilitate decentralized, point-of-care manufacturing. A phenotypic and functional comparison of manually and robotically manufactured products was outside the scope of this study. Such analyses were reported previously (Melocchi et al., 2024), and a complete characterization of the final product is planned once a fully integrated end-to-end workflow is in place. Finally, the platform has not yet been evaluated for adherent cells or for stem-cell products such as induced pluripotent stem cells. Extending the platform to these workflows is a promising avenue that will require dedicated development and validation.

## 5 Conclusions

Robotic automation has the potential to perform many manual procedures in cell therapy manufacturing. End-to-end automation of a modular manufacturing process may increase the supply of cell therapy products to meet global demand, improve efficiency, accelerate delivery timelines, and ensure high quality standards are consistently met.

The following are the supplementary data related to this article.

Supplementary video 1 Video showing the robotic workflow for each unit operation.

Supplementary material 1

## CRediT authorship contribution statement

**Sudeshna Sadhu:** Writing – original draft, Visualization, Investigation, Formal analysis, Data curation. **Brigitte Schmittlein:** Writing – original draft, Visualization, Investigation, Formal analysis, Data curation. **Angela Lares:** Writing – original draft, Supervision, Data curation. **Christopher Cheng:** Writing – review & editing, Writing – original draft, Visualization. **Xiaojie Chen:** Writing – review & editing, Visualization. **Varun Bhatia:** Writing – review & editing, Visualization. **Winston Zha:** Writing – review & editing, Visualization. **Sebastien Wah:** Writing – review & editing. **Sunaina Nayak:** Investigation. **Marco Uboldi:** Investigation. **Yifan Yu:** Resources. **Jiawei Wang:** Resources. **Wei Zhang:** Resources. **Prajakta P. Bhanap:** Resources. **Yongchang Ji:** Resources. **Andrew Scheffler:** Resources. **John Wilson:** Resources. **Dan Welch:** Resources. **Nikolaos Gkitsas-Long:** Investigation. **Aidan Jeffrey Retherford:** Investigation. **Ramya Tunuguntla:** Investigation. **Alice Melocchi:** Writing – original draft, Visualization, Supervision, Data curation. **Alexandre Girard:** Writing – review & editing, Conceptualization. **Federico Parietti:** Supervision, Funding acquisition, Conceptualization. **Steven A. Feldman:** Writing – review & editing, Supervision, Conceptualization. **Jonathan H. Esensten:** Writing – review & editing, Supervision, Data curation, Conceptualization.

## Funding

This work was supported in part through a collaboration between Stanford Center for Cancer Cell Therapy and Multiply Labs, Inc.

Data availability

All data are available in the main text or in the supplementary materials.

## Declaration of competing interest

The authors declare the following financial interests/personal relationships which may be considered as potential competing interests: Sudeshna Sadhu, Brigitte Schmittlein, Angela Lares, Christopher Cheng, Xiaojie Chen, Varun Bhatia, Winston Zha, Sebastien Wah and Sunaina Nayak wish to disclose that they are current employees of Multiply Labs, Inc. or were employed with the company at the time of this study's execution. They hold equity in the company; Alice Melocchi and Federico Parietti wish to disclose that they are co-founders of Multiply Labs, Inc. and hold the position of Chief Scientific Officer and Chief Executive Officer, respectively; Yifan Yu, Jiawei Wang, Wei Zhang wish to disclose that they are employed by GenScript Biotech Corporation, which is a company active in the cell therapy manufacturing space; Prajakta P. Bhanap and Yongchang Ji wish to disclose that they are employed by Thermo Fisher Scientific, which is a company active in the cell therapy manufacturing space; Andrew Scheffler wishes to disclose that he is employed by ScaleReady, which is a company active in the cell therapy manufacturing space; John Wilson and Dan Welch wish to disclose that they are employed by Wilson Wolf Manufacturing corporation, which is a company active in the cell therapy manufacturing space; Nikolaos Gkitsas-Long, Aidan Jeffrey Retherford, Ramya Tunuguntla and Steven A. Feldman wish to disclose that they work in the Laboratory for Cell and Gene Therapy Manufacturing (LCGM) at Stanford University. The LCGM entered a collaboration agreement with Multiply Labs, Inc. for studying the performance of cell therapy manufacturing robots built by the company, by comparing them with manual processes; Jonathan H. Esensten wishes to disclose he is a paid advisor for Multiply Labs, Inc. He serves on its scientific advisory board and holds equity in the company. He receives sponsored research funding from Lonza, Inc. for the development of cellular therapy manufacturing devices.

## Data Availability

Data will be made available on request.
