# Supplementary material for: Performance of a modular robotic cluster matches skilled human operators for complex cell therapy manufacturing tasks
Source: Int J Pharm X. 2026 Aug 4;12:100631. doi: 10.1016/j.ijpx.2026.100631 (PMC13487720; doi:10.1016/j.ijpx.2026.100631)
Supplement: Supplementary file 1 [file mmc2.docx]

**Supplementary Materials**

**Figure S1:** Percentage of different cell populations post-selection were assessed in the positive fraction after CD4 and CD8 cell selection on CytoSinct 1000 using flow cytometry (n = 3).

**Figure S2.** Automation strategy for CTS Rotea counterflow centrifugation instrument. a) Image of off-the-shelf instrument used in manual workflow. b) off-the-shelf standard consumable used in manual cell selection workflow. c) Cartridge to assemble standard consumable for automated process D) 3D render showing different components for instrument automation: robotic arm interacting with robotic cartridge and off-the-shelf instrument with dock plate to house robotic cartridge.

**Video S1:** Video showing the robotic workflow for each unit operation.

**Table S1:** Hardware and software design specifications for the robotic cluster. Orange = future/planned improvements (for future iteration of robotic systems).

| **User Requirement** | **SW features** | **HW features** |
| --- | --- | --- |
| **Modularity**  *clusters can contain different sets of instruments* | - Industrial equipment is operated remotely using protocols like Modbus TCP (the company standard) and OPC UA, with most in-house software built around Modbus. - Some instruments (*e.g.* Rotea, CytoSinct, NC-200) rely on their own APIs; which are hosted on virtual machines (VMs) running on OnLogic devices and bridged to Modbus/OPC UA. - When vendors only provide GUI interfaces, APIs or TCP/IP protocols are requested for automation and remote control. - Simulation tools like RoboDK test the robotic feasibility of new module layouts, ensuring EOAT access to cartridges/docking stations, safe robotic arm orientation within operating limits, and no collisions between arms, walls, or other cluster components. - Automated consumable location assignment within modules: when a process is created, it includes variables that define where consumables need to be located at different time points. Once the cluster layout is set, the system can automatically assign the appropriate locations based on those variables. - Containerized SW on edge devices for cloud to module level communication: instead of deploying a single, monolithic system, we break it down into modular software components which mirrors the hardware modularity. | - Modules are removable and swappable within a given cluster configuration. - The system automates a variety of instruments to accomplish every major task in the cell manufacturing process. - Needlefree and Texium connectors allow for on-demand connections, and multiple connections/disconnections per consumable line. |
| **Flexibility**  *system supports process changes before and during run* | - HMI (human machine interface) and live inputs allow user to view the run steps in real time and specify certain parameters (like reagent volumes) mid-process - The planner takes a process and generates a plan for a specific cluster. This plan is generated offline and before the run. The orchestrator executes the plan: instructing modules to begin execution at the correct times, and reacting if things don't go according to plan. - The process creator app (PCA) is a no-code interface for customizing order of steps or smaller parameters; for example: reagent volumes, number of resuspensions, incubation times. - Replanning: the robotic system plan can change based on cell counts and other variables received mid-process (would need to reconstruct the cluster state and use that to plan from arbitrary cluster states). | - The system automates a variety of syringe, vial, and bag sizes. |
| **Comparability**  *bio process does not differ too much from a manual one so that tech transfer is easier/ faster and bio success criteria are met* | - The “constraint solver” planning software ensures that biological time constraints are met during a process. - A scientist’s manual movements (*e.g.* bioreactor resuspension) can be recorded and copied *via* a vision system (OptiTrak). | - Minimal modification to equipment (*e.g.* Rotea, CytoSinct, NC-200). - Minimal modification to consumables (add needlefree/ Texium connectors, but materials remain the same). |
| **Error recovery**  *system automatically detects and recovers from errors. This section also includes features we’ve implemented to reduce the risk of errors* | - Error/retry logic: various sensors detect when, for example, an expected force limit hasn't been reached. If the system detects an error, it pauses the run and prompts the operator to resolve the issue. The operator can then either resume with retry or without retry. - Auto calibration system and force mode are used to increase system robustness: methods include vision system, ToF sensor, and manual calibration. - Power loss robustness: the system doesn’t currently have full power loss robustness, but does have a database to store the operation data for what's already occurred so that the data isn’t lost. | - Torque sensors on EOATs to detect whether a successful connection has been made. - Mechanical locating features on docking stations for coarse and precise alignment (increases system robustness to cartridge pick/place misalignments). |
| **Sterility**  *cells are manufacturing in aseptic conditions* | - N/A | - Needlefree/Texium connectors and disinfecting caps create a functionally closed connection system. - Process stream contacting components are single use. - HEPA filters above each module creates a grade C environment (included in the system, but not used during the runs). |
| **Throughput**  *system designed to increase throughput by running multiple processes/batches at once* | - Load/unload wave allocation for consumables makes manual interventions quick and simple. - Single arm parallelism: a single arm can switch between different tasks or batches when one task is taking a long time. - Multiple arm parallelism: same concept as single arm parallelism, but taking multiple robotic arms within the same system into account. | - 18 standard 1L bioreactors per incubator allows for many products to be processed in parallel. - Each module is designed to contain the max number of items (docking stations, cartridges) needed for a given process. |
| **Safety**  *user interaction with system/arm is safe* | - Collaborative arm: the robots have in-built force feedback to detect if a collision has occurred and stop any motion. - Arm collision avoidance system: sensors to determine when an arm is too close to another arm and pause motion (only relevant for >1 arm systems). | - Emergency stop to completely halt the system if needed. - Dead man’s switch for robotic arm when personnel enters the cluster (*i.e.* to calibrate the robot), will completely pause arm movement. - Cluster doors with electronic interlocks that can be accessed via the HMI. |
| **Security**  *company/process data is secure* | - HMI and PCA support different levels of user access. - Single tenant cloud architecture - each customer gets a dedicated AWS account to isolate customer data from other customer data. - Compliant with certain cyber security measures (especially for remote monitoring and access). | - N/A |
| **Traceability**  *process steps, errors, etc. are recorded* | - Digital record generated after each run that lists process steps, timing, errors, and other process-specific parameters. - Barcode scanning on consumables/cartridges tracks movement through the system. | - Cameras allow for remote visualization of modules during runs. |
| **Sourcing / supply chain**  *components are easily sourced / exchanged* | - N/A | - Consumables strategy enables minimal changes from existing off the shelf consumable vials, bags, syringes, and tubing kits. - HW is off the shelf wherever possible (not custom designed). |
| **Autonomy**  *minimal manual interventions* | - The system enables robotic calculations & automated decisions based on cell counts and other instrument or user inputs. | - Input/output module is the only one where a human/operator would interact. |
| **Cleaning/Maintenance** | - N/A | - The system can be accessed via an outer door, cluster modules also have doors. - All modules and docking stations are compatible with common cleaning agents, are IP54, and have cleanable external features (*e.g.* no gaps). |

| **System functions** | | |
| --- | --- | --- |
| **Fluid transfer**  *transfer of fluid volumes accurately (+/-5% for large volumes, +/-10% for small volumes)* | - Closed loop pumping (based on weight data) between bags and bioreactors. - Software algorithms estimate flow rates in order to ensure fluid transfer accuracy. | - Weigh scales below bag stations track weights and inform closed loop pumps. - Pump system design is optimized for accuracy (*e.g.* number of rollers, tube diameter and materials, etc.) - Coiled tubes are used to make between consumables across long distances and avoid line kinks during pumping. |
| **Reagent preparation and bioreactor sampling**  *syringe/vial interactions (automated syringe push/pull and vial draw/dispense)* | - Sample volumes can be input mid-process. | - Electromagnets on docking stations securely hold cartridges or bioreactors during sampling or reagent preparation steps. - Syringe EOAT enables syringe plunger actuation. |
| **Gas control**  *CO_2_ to incubators* | - Gas concentrations are recorded in graph format in the digital record. - Alarms when gas concentration is out of specification. | - The gas manifold system allows transfer to all relevant modules. |
| **Temperature control** *bioreactors and culture bags can be incubated and reagents can be stored in 4°C environment* | - External temperature sensors within the incubator and refrigerator to monitor levels and alarm if out of specification. | - Automated incubator module with sliding custom drawers with bioreactor slots/docks - Automated fridge and media warmer modules |
| **CTS Rotea Counterflow centrifugation system**  *automated kit installation and protocol generation/run* | - SW control via OPCUA communication protocol | - Robotic tube handling and connection - Location and locking of cartridge/consumable onto equipment - Sampling from cell input and harvest bags |
| **CytoSinct 1000**  *automated kit installation and protocol generation/run* | - SW control via custom API | - Tube handling and connection - Location and locking of cartridge/consumable onto equipment - Sampling from cell input and harvest bags |
| **NucleoCounter NC-200**  *automated cell counting* | - SW control via custom API | - Station for aspirating cell sample - Microfluidic chamber for transferring sample into Via1 cassette - Placement of cassette into NC-200 slot using designated EOAT/force mode |

**Table S2:** metrics assessed for each unit operation performed manually and robotically.

| **Unit Operations** | **Parameters** | **Mean ± SD (n)** | |
| --- | --- | --- | --- |
|  |  | **Manual Condition** | **Robotic Condition** |
| **Vial-to-syringe draw** | Draw Volume | 5.92 mL ± 0.05 (n=21) | 5.97 mL ± 0.03 (n=21) |
|  | Accuracy of draw | 102.4 % ± 0.93 (n=21) | 103 % ± 0.60 (n=21) |
| **Bag Resuspension and Sampling** | Viable cell counts | 1.53x10^9^ cells ± 2.00 x10^9^ cells (n=6) | 1.50 x 10^9^ cells ± 1.96 x 10^9^ cells (n=6) |
| **Bioreactor Resuspension and Sampling** | High volume, low concentration: Viable cell concentration | 7.54 x10^4^ cells/mL ± 1.78 x10^4^ cells/mL (n=3) | 8.78 x10^4^ cells/mL ± 0.71 x10^4^ cells/mL (n=3) |
|  | Low volume, low concentration: Viable cell concentration | 4.68 x10^5^ cells/mL ± 0.40 x10^5^ cells/mL (n=3) | 5.91 x10^5^ cells/mL ± 1.02 x10^5^ cells/mL (n=3) |
|  | High volume, high concentration: Viable cell concentration | 2.85 x10^6^ cells/mL ± 0.48 x 10^6^ cells/mL (n=3) | 3.59 x10^6^ cells/mL ± 0.25 x10^6^ cells/mL (n=3) |
|  | Low volume, high concentration: Viable cell concentration | 1.13 x 10^7^ cells/mL ± 0.44 x 10^7^ cells/mL (n=3) | 1.37 x 10^7^ cells/mL ± 0.47 x 10^7^ cells/mL (n=3) |
| **Cell count** | High concentration: Viable cell concentration | 3.73 x 10^6^ cells/mL ± 0.12 x 10^6^ cells/mL (n=15) | 3.69 x 10^6^ cells/mL ± 0.10 x 10^6^ cells/mL (n=15) |
|  | Low concentration: Viable cell concentration | 5.90 x 10^5^ cells/mL ± 0.14 x 10^5^ cells/mL (n=15) | 5.63 x 10^5^ cells/mL ± 0.25 x 10^5^ cells/mL (n=15) |
| **Cell selection** | T cell purity | 88.88 % ± 5.04 (n=5) | 90.47 % ± 4.92 (n=6) |
|  | Cell viability | 90.87 % ± 2.28 (n=5) | 89.18 % ± 3.68 (n=6) |
|  | Cell Recovery | 44.85 % ± 16.38 (n=5) | 50.47 % ± 35.73 (n=6) |
| **Buffer exchange and cell harvest** | Cell Recovery | 90.64 % ± 5.28 (n=4) | 102.3 % ± 27.14 (n=3) |
|  | Post-harvest cell viability | 94.33 % ± 0.91 (n=4) | 89.11 % ± 6.88 (n=3) |

**Table S3:** Aseptic process simulation results using robotic unit operations for a generic effector T cell manufacturing process.

| **Experiment types** | **Runs** | **14-day sterility test (Growth / No Growth)** | **Pass / Fail** |
| --- | --- | --- | --- |
| **Media fill** | 1 | No growth | **Pass** |
|  | 2 | No growth | **Pass** |
|  | 3 | No growth | **Pass** |
|  | 4 | No growth | **Pass** |

**Table S4:** manual and robotic consumables used in each unit operation.

| **Unit Operations** | **Consumables and robotic cartridges** | **Consumables *per* operation** | |
| --- | --- | --- | --- |
|  |  | **Manual Condition** | **Robotic**  **Condition** |
| **Vial-to-syringe draw** | Vial | 1 | 1 |
|  | 10 mL BD syringe | 1 | 1 |
|  | Vial spike / adaptor | 1 | 1 |
|  | SmartSite connectors with robotic port | 0 | 1 |
|  | Texium connectors with robotic coupler | 0 | 1 |
|  | Disinfecting caps | 0 | 2 |
|  | Robotic vial adaptor | 0 | 1 |
| **Bag-to-bag transfer** | Bags | 2 | 2 |
|  | Transfer sets | 2 | 0 |
|  | SmartSite connectors with robotic port | 0 | 2 |
|  | Texium connectors with robotic coupler | 0 | 2 |
|  | Robotic tubings (coiled tube, pump tube, medical grade barbs) | 0 | 2 |
|  | Disinfecting caps | 0 | 2 |
|  | Robotic bag cartridges | 0 | 2 |
| **Bag Resuspension and Sampling** | Bags | 1 | 1 |
|  | Transfer sets | 1 | 0 |
|  | 10 mL BD syringe | 0 | 1 |
|  | Sampling pouch | 1 | 0 |
|  | Texium connectors with robotic coupler | 0 | 2 |
|  | SmartSite connectors with robotic port | 0 | 1 |
|  | Robotic tubings (coiled tube, pump tube, medical grade barbs) | 0 | 1 |
|  | Disinfecting caps | 0 | 2 |
|  | Robotic bag cartridges | 0 | 1 |
| **Bioreactor Resuspension and Sampling** | G-Rex bioreactor | 1 | 0 |
|  | G-Rex bioreactor with robotic adaptor | 0 | 1 |
|  | 10 mL BD syringe | 0 | 1 |
|  | Sampling pouch | 1 | 0 |
|  | SmartSite connectors with robotic port | 0 | 3 |
|  | Texium connectors with robotic coupler | 0 | 1 |
|  | Disinfecting caps | 0 | 2 |
| **Cell count** | Via-1 cassette | 1 | 1 |
|  | 10 mL BD syringe | 0 | 1 |
|  | Eppendorf / conical tubes | 1 | 0 |
|  | Micropipette and tips | 1 | 0 |
|  | SmartSite connectors with robotic port | 0 | 1 |
|  | Robotic cell counting cassette + tubing | 0 | 1 |
| **Cell isolation** | CytoSinct isolation kit | 1 | 0 |
|  | Robotic CytoSinct isolation kit | 0 | 1 |
|  | Bags | 6 | 6 |
|  | 10 mL BD syringe | 0 | 1 |
|  | SmartSite connectors with robotic port | 0 | 12 |
|  | Texium connectors with robotic coupler | 0 | 7 |
|  | Robotic tubings (coiled tube, pump tube, medical grade barbs) | 0 | 6 |
|  | Disinfecting caps | 0 | 14 |
|  | Robotic CytoSinct cartridge | 0 | 1 |
|  | Robotic bag cartridges | 0 | 6 |
| **Buffer exchange and cell harvest`** | Rotea kit | 1 | 1 |
|  | Robotic Rotea kit | 0 | 1 |
|  | Sampling pouch | 1 | 0 |
|  | Bags | 8 | 8 |
|  | 10 mL BD syringe | 0 | 1 |
|  | SmartSite connectors with robotic port | 0 | 16 |
|  | Texium connectors with robotic coupler | 0 | 9 |
|  | Robotic tubings (coiled tube, pump tube, medical grade barbs) | 0 | 8 |
|  | Disinfecting caps | 0 | 17 |
|  | Bag cartridge | 0 | 8 |
|  | Robotic Rotea cartridge | 0 | 1 |
